# A Novel and Cross-Species Active Mammalian INDY (NaCT) Inhibitor Ameliorates Hepatic Steatosis in Mice with Diet-Induced Obesity

**DOI:** 10.3390/metabo12080732

**Published:** 2022-08-08

**Authors:** Grit Zahn, Diana M. Willmes, Nermeen N. El-Agroudy, Christopher Yarnold, Richard Jarjes-Pike, Sabine Schaertl, Kay Schreiter, Wiebke Gehrmann, Andrea Kuan Cie Wong, Tommaso Zordan, Jörg König, Jens Jordan, Andreas L. Birkenfeld

**Affiliations:** 1 Eternygen GmbH, Müllerstrasse 178, 13353 Berlin, Germany; 2 Department of Diabetology Endocrinology and Nephrology, Internal Medicine IV, University Hospital Tübingen, Eberhard Karls University Tübingen, 72074 Tübingen, Germany; 3 Section of Metabolic and Vascular Medicine, Medical Clinic III, Dresden University School of Medicine, Technische Universität Dresden, 01069 Dresden, Germany; 4 Division of Translational Diabetology, Institute of Diabetes Research and Metabolic Diseases (IDM) of the Helmholtz Center Munich, Eberhard Karls University Tübingen, 72074 Tübingen, Germany; 5 Department of Diabetes, School of Life Course Science and Medicine, Kings College London, London WC2R 2LS, UK; 6 Evotec (UK) Ltd., Oxfordshire OX14 4RZ, UK; 7 Evotec SE, 22419 Hamburg, Germany; 8 Evotec International GmbH, 37079 Göttingen, Germany; 9 Aptuit (Verona) Srl, an Evotec Company, 37135 Verona, Italy; 10 Institute of Experimental and Clinical Pharmacology and Toxicology, Friedrich-Alexander-Universität Erlangen-Nürnberg, Fahrstrasse 17, 91054 Erlangen, Germany; 11 German Aerospace Center (DLR), Institute of Aerospace Medicine, 51147 Cologne, Germany

**Keywords:** MAFLD, NASH, NAFLD, mINDY, SLC13A5, citrate transporter, NaCT, metabolic dysfunction

## Abstract

Mammalian INDY (mINDY, NaCT, gene symbol *SLC13A5*) is a potential target for the treatment of metabolically associated fatty liver disease (MAFLD). This study evaluated the effects of a selective, cross-species active, non-competitive, non-substrate-like inhibitor of NaCT. First, the small molecule inhibitor ETG-5773 was evaluated for citrate and succinate uptake and fatty acid synthesis in cell lines expressing both human NaCT and mouse Nact. Once its suitability was established, the inhibitor was evaluated in a diet-induced obesity (DIO) mouse model. DIO mice treated with 15 mg/kg compound ETG-5773 twice daily for 28 days had reduced body weight, fasting blood glucose, and insulin, and improved glucose tolerance. Liver triglycerides were significantly reduced, and body composition was improved by reducing fat mass, supported by a significant reduction in the expression of genes for lipogenesis such as *SREBF1* and *SCD1*. Most of these effects were also evident after a seven-day treatment with the same dose. Further mechanistic investigation in the seven-day study showed increased plasma β-hydroxybutyrate and activated hepatic adenosine monophosphate-activated protein kinase (AMPK), reflecting findings from *Indy* (−/−) knockout mice. These results suggest that the inhibitor ETG-5773 blocked citrate uptake mediated by mouse and human NaCT to reduce liver steatosis and body fat and improve glucose regulation, proving the concept of NaCT inhibition as a future liver treatment for MAFLD.

## 1. Introduction

Fatty liver associated with metabolic dysfunction is common and affects around one billion individuals worldwide [1,2]. The disease, so far known as non-alcoholic fatty liver disease (NAFLD), was recently renamed metabolic dysfunction associated fatty liver disease—MAFLD—to better reflect the patient heterogeneity and pathogenesis [3,4]. MAFLD may progress to non-alcoholic steatohepatitis (NASH), which is characterised by hepatic inflammation and fibrosis [5], and predisposes patients to cirrhosis and hepatocellular carcinoma (HCC) [6]. Although cirrhosis and HCC are serious and potentially lethal diseases, most patients with MAFLD die from cardiovascular disease or complications of type 2 diabetes mellitus. Indeed, hepatic fibrosis independently predicts all-cause mortality [7]. Because central obesity, type 2 diabetes mellitus, dyslipidaemia, and insulin resistance promote MAFLD [8], lifestyle interventions leading to body weight loss ameliorate MAFLD [9]. However, body weight often relapses such that lifestyle interventions, while being beneficial, may not suffice in controlling MAFLD in the long term [10]. There is a need for targeted pharmacological therapies for MAFLD.

Solute carrier family 13 member 5 (*SLC13A5*, *NaCT*), the mammalian orthologue of the I’m not dead yet (*Indy*) gene in Drosophila melanogaster [11,12], is a promising treatment target for MAFLD and associated cardiometabolic diseases. In mammals, the NaCT protein is a tricarboxylate plasma membrane transporter with a preference for citrate. *Indy* (Nact) knockout in mice protected high-fat-diet-fed and aged animals from adiposity and insulin resistance [13] and lowered blood pressure through sympathetic inhibition [14]. Antisense knockdown in high-fat-diet-fed rats reduced fasting insulin concentrations, plasma triglycerides, and hepatic triglyceride levels [15]; beneficial effects on hepatic triglycerides were confirmed in an siRNA study in mice [16]. In human hepatocytes, *SLC13A5* silencing reduced overall lipid content [17]. Importantly, hepatic NaCT expression was increased in patients with type 2 diabetes mellitus, MAFLD, and obesity [18,19]. In patients with MAFLD, NaCT protein expression was independently associated with the degree of hepatic steatosis [18]. Therefore, compounds inhibiting NaCT have been sought for in recent years. Some early compounds showed weak in vitro activity [20,21] or were only effective in cells [22]. Substrate-like NaCT inhibitors (e.g., PF-06649298) blocked citrate uptake in hepatocytes and mice [23,24]. Further optimisation improved their activity but worsened their selectivity, which resulted in side effects such as increased urinary calcium secretion [23,24].

In this study, we characterise a highly selective, cross-species active, non-competitive, non-substrate inhibitor (ETG-5773). We profiled ETG-5773 by in vitro testing in human hepatocytes and cell lines recombinantly expressing the transporter and in a mouse model of diet-induced obesity (DIO). In mice, we hypothesised that ETG-5773 is able to reduce liver fat and other features of MAFLD.

## 2. Results

### 2.1. Selection of the Inhibitor

**Figure 1:**
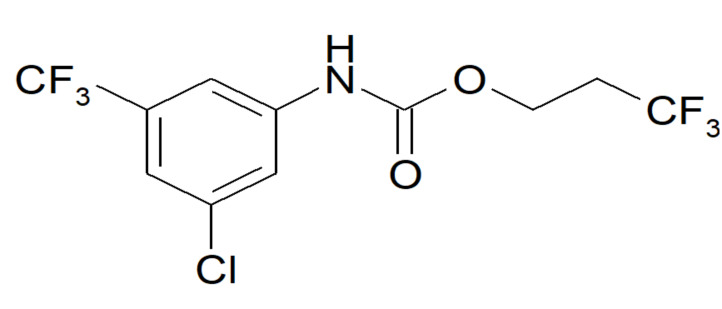
Molecular structure of the ETG-5773 compound.

We screened a library containing more than 50,000 highly diverse lead-like compounds and fragments. We applied a functional citrate uptake assay with [14C] citrate in HEK293 cells overexpressing recombinant human NaCT. To confirm the activity of the selected compounds on NaCT, we measured citrate uptake in HepG2 cells, which endogenously expressed human NaCT. Furthermore, we tested their selectivity in vitro in different cell lines expressing other non-related transporters (e.g., GLT1, GLUT1). This approach, following hit identification, hit expansion, and hit-to-lead optimisation, resulted in the selection of ETG-5773 as a compound of interest with selective inhibition of NaCT (Figure 1).

### 2.2. In Vitro Activity and Selectivity of ETG-5773

To test the inhibitory activity of ETG-5773 on the NaCT transport function, we assessed the radioactive citrate uptake in human and in murine cells expressing the target protein. We measured the human transport activity in HepG2 cells and the activity on the mouse transporter in HEK293 cells recombinantly overexpressing Nact. As shown in Table 1, ETG-5773 reduced citrate uptake in the nanomolar range in cell lines expressing mouse or human NaCT, with an IC50 of 160nM for human, and 180nM for mouse Nact, demonstrating the cross-species activity of the inhibitor. An exemplary inhibition profile of citrate uptake is shown in Figure S2. To analyse the transporter specificity, we also tested the compound for its activity on the closely related human NaDC3 (SLC13A3) transporter with succinate uptake assays in HEK293 cells overexpressing NaDC3. ETG-5773 showed no activity at concentrations up to 20 µM, indicating, at the least, a more than 100-fold selectivity.

To establish whether reduced citrate uptake was reflected in the degree of citrate-mediated fatty acid synthesis, we measured [14C] the citrate incorporation in HepG2 cells. The results showed that the inhibitory activity in citrate uptake assays translates to an inhibitory activity on fatty acid synthesis. A parallel assessment of viability showed no cytotoxic side effects at concentrations up to 50 µM (Table 1).

Furthermore, we directly compared ETG-5773 activity on NaCT-mediated uptake to known inhibitors as shown in Table 2. In comparison to PF-06649298, a substrate-like inhibitor with activity towards both mouse and human NaCT [23], ETG-5773 was active at much lower concentrations, affecting citrate uptake in the nanomolar range rather than the micromolar range for both species. This finding was supported by an around 40-fold IC_50_ difference in fatty acid synthesis. Compared to BI01383298, an inhibitor that irreversibly binds to NaCT [22], ETG-5773 had a similar if slightly lower activity for human NaCT as indicated by citrate uptake and fatty acid synthesis (FAS). However, BI01383298 is considered to be human specific and showed no effects on citrate uptake for mouse Nact.

### 2.3. In Vivo Investigation of ETG-5773 in a Mouse Model of Diet-Induced Obesity

Next, we tested the functional activity of ETG-5773 in vivo to establish whether the effects on in vitro citrate uptake and FAS were physiologically relevant. Preliminary short-term tolerability studies in the DIO mouse model showed a reduced food intake, which was not observed in *Indy* knockout mice. Therefore, we applied pair-fed control groups to explore the pharmacological actions on metabolism without interference from food-intake effects.

**Figure 2:**
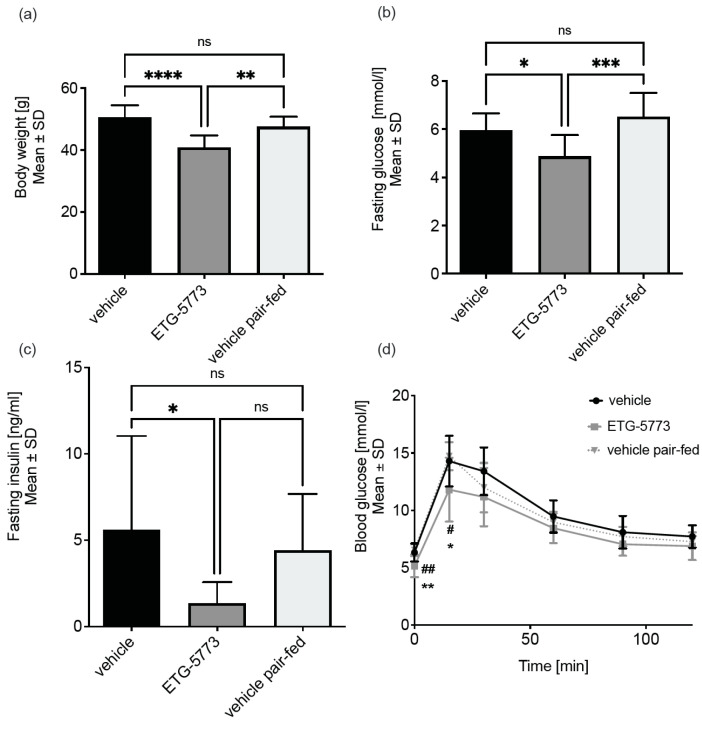
Treatment with ETG-5773 changes metabolic parameters in obese mice. (**a**) Body weight of DIO mice; (**b**) fasting blood glucose levels; (**c**) fasting serum insulin levels; * *p* < 0.05, ** *p* < 0.01, *** *p* < 0.001, **** *p* < 0.0001; (**d**) glucose tolerance test, # ETG-5773 vs. vehicle, * ETG-5773 vs. vehicle pair-fed, * or # *p* < 0.05, ** or ## *p* < 0.01, ns: non-significant, ANOVA, mean SD. Animals were treated orally twice a day with 15 mg/kg ETG-5773 or with the corresponding vehicle or vehicle in the pair-fed group, *n* = 12 vehicle and ETG-5773, *n* = 8 pair-fed.

***28-Day treatment with ETG-5773 ameliorates diet-induced obesity.*** In DIO mice (*n* = 12 vehicle and ETG-5773, *n* = 8 pair-fed) treated orally with 15 mg/kg twice-daily ETG-5773, we observed a 14% reduction in body weight compared to the pair-fed group (*p* < 0.01) and a 19% reduction compared to the vehicle group (*p* < 0.0001, Figure 2a). Fasting blood glucose levels were significantly reduced by 25% (*p* < 0.001) and fasting insulin concentrations were numerically reduced by 70% (non-significant, *p* = 0.2) in the treated mice compared to the pair-fed mice (Figure 2b,c). Comparison of the ETG-5773-treated animals with the vehicle-treated mice showed a 17% (*p* < 0.05) decrease in fasting blood glucose concentrations and a 75% (*p* < 0.05) reduction in fasting insulin. Intraperitoneal glucose tolerance testing (IPGTT) indicated significantly improved glucose tolerance in the treated group compared to the vehicle control and the pair-fed group (all *p* < 0.05, Figure 2d). Further analysis of the overall glucose excursion curve showed a significantly reduced area under the curve (AUC) compared to the vehicle control (*p* < 0.05) but this was not significant compared to the pair-fed control.

**Figure 3:**
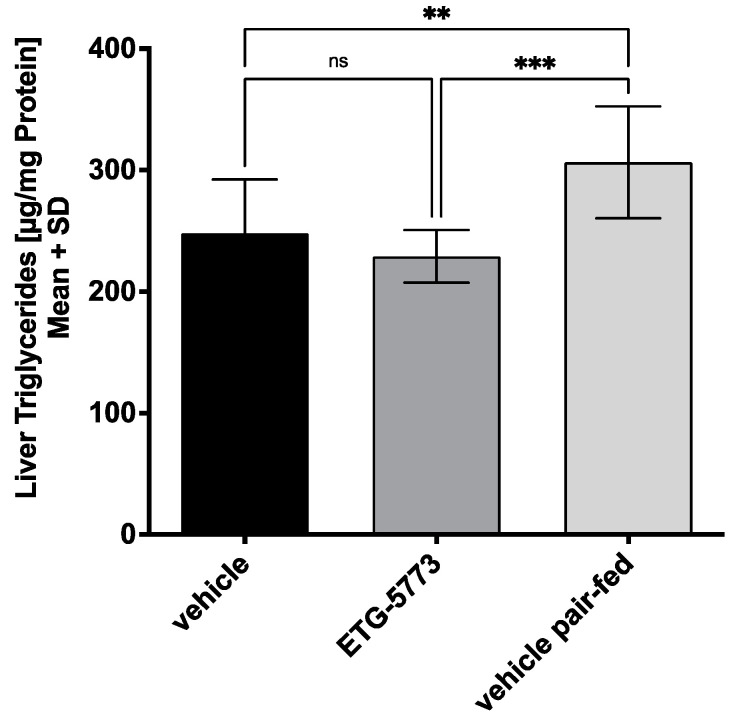
Liver triglycerides are reduced in obese mice treated with ETG-5773. Triglyceride content in the livers of DIO mice after the 28-day treatment period. Animals were treated orally twice a day with 15 mg/kg ETG-5773 or with the corresponding vehicle or vehicle in the pair-fed group ** *p* < 0.01, *** *p* < 0.001, ns: non-significant, ANOVA mean SD, *n* = 12 vehicle and ETG-5773, *n* = 8 pair-fed.

***28-Day Treatment with ETG-5773 attenuates hepatic steatosis***. High-fat-diet feeding leads to an increased hepatic triglyceride content as one component of MAFLD. The ETG-5773 15 mg/kg treatment for 28 days reduced the hepatic triglyceride content by 25% compared to the pair-fed animals (*p* < 0.001) but not compared to the vehicle (Figure 3). This result suggests that the ETG-5773 treatment was beneficial in treating hepatic steatosis. Plasma lipoprotein analyses showed no effect on the cholesterol concentrations. However, the plasma triglyceride concentrations were slightly increased compared to the pair-fed animals (*p* < 0.01), though not compared to the vehicle (Figure S3).

**Figure 4:**
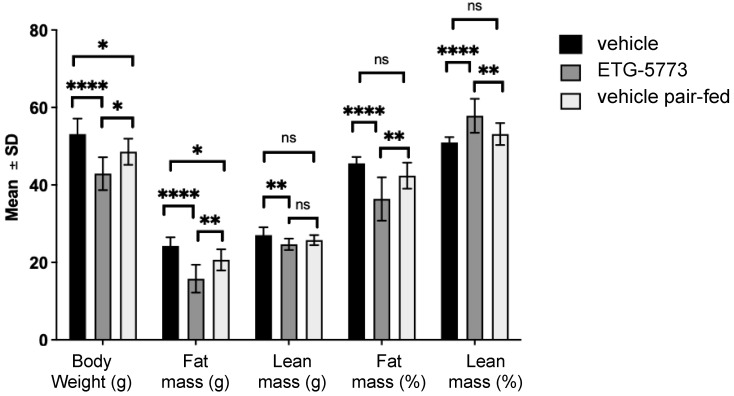
Body composition analysis in DIO mice treated with ETG-5773. The graph shows changes in absolute body weight, fat mass, and lean mass of mice after 22 days of treatment as well as relative amounts of fat and lean mass. Animals were treated orally twice a day with 15 mg/kg ETG-5773 or with the corresponding vehicle for vehicle or pair-fed groups. * *p* < 0.05, ** *p* < 0.01, **** *p* < 0.0001, ns: non-significant. ANOVA mean SD, *n* = 12 vehicle and ETG-5773, *n* = 8 pair-fed.

***[1H]-Nuclear Magnetic Resonance Analysis of Body Composition.*** We applied proton-nuclear magnetic resonance (NMR) to analyse the body composition in vivo (Figure 4). In the ETG-5773 15 mg/kg treated animals, fat mass was markedly reduced at day 22 compared to both the vehicle control (*p* < 0.0001) and the pair-fed group (*p* < 0.01), while the lean mass in treated mice was only significantly lower than the vehicle-treated control group (*p* < 0.01). When we calculated fat and lean mass as percentages of body weight, there was a significant decrease in body fat and a significant increase in lean mass compared to both the vehicle (*p* < 0.0001) and pair-fed control groups (all *p* < 0.01). Changes in lean mass, which comprises muscle mass and bone, among others, are largely mediated by muscle mass.

**Figure 5:**
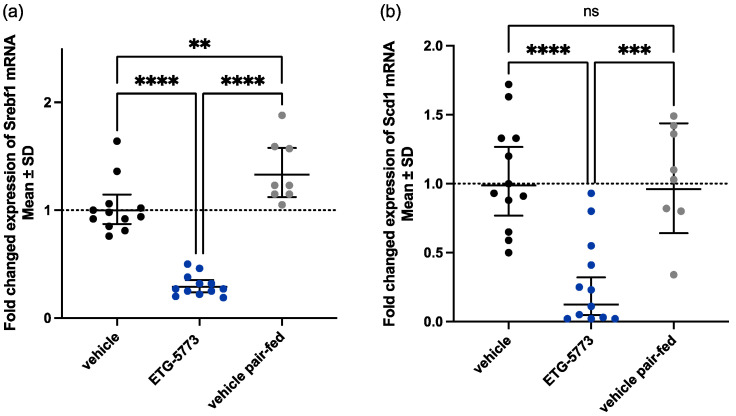
Hepatic mRNA expression levels of key genes involved in lipogenesis in DIO mice after the 28-day treatment period. Animals were treated orally twice a day with ETG-5773 at 15 mg/kg or with the corresponding vehicle or vehicle in the pair-fed group: (**a**) mRNA expression of sterol regulatory element-binding transcription factor 1 (*Srebf1*); (**b**) mRNA expression of the stearoyl-CoA desaturase (*Scd1*) gene. ** *p* < 0.01, *** *p* < 0.001, **** *p* < 0.0001 as indicated, ns: non-significant. ANOVA mean SD, *n* = 12 vehicle and ETG-5773, *n* = 8 pair-fed.

***28-Day treatment with ETG-5773 regulates lipogenesis-related genes.*** Liver fat accumulation in obese mice is driven by the uptake of circulating fatty acids but also by *de novo* lipogenesis. To assess the effects of the ETG-5773 treatment on the regulation of *de novo* lipogenesis, we analysed the expression of selected genes in the lipogenesis pathways, such as sterol regulatory element-binding protein 1 (*Srebf1*), a transcriptional lipogenesis master regulator, as well as downstream enzymes (Figure 5). We observed the strongest downregulation for *Srebf1* and Stearoyl-CoA desaturase (*Scd1*), the enzyme catalysing the rate-limiting step in the formation of monounsaturated fatty acids (MUFAs). Other genes regulated by Srebf1, such as acetyl coenzyme A carboxylase 1 (*Acc1*), *Acly*, and fatty acid synthase (*Fasn*) were downregulated as well (Figure S4). *Srebf1* mRNA was significantly downregulated in mice treated with 15 mg/kg ETG-5773 compared to both the vehicle control and the pair-fed group (*p* < 0.0001, Figure 5a). *Scd1* mRNA levels were significantly downregulated compared to the vehicle control (*p* < 0.0001) and the pair-fed group (*p* < 0.001, Figure 5b). *Acly* mRNA levels were significantly downregulated compared to the vehicle control (*p* < 0.001) and the pair-fed group (*p* < 0.01, Figure S4a) and Acc1 and Fasn mRNA levels were significantly downregulated compared to the vehicle control (*p* < 0.05 and *p* < 0.01) and showed a trend for reduction compared to the pair-fed group (*p* < 0.1 and 0.09, Figure S4b,c).

**Figure 6:**
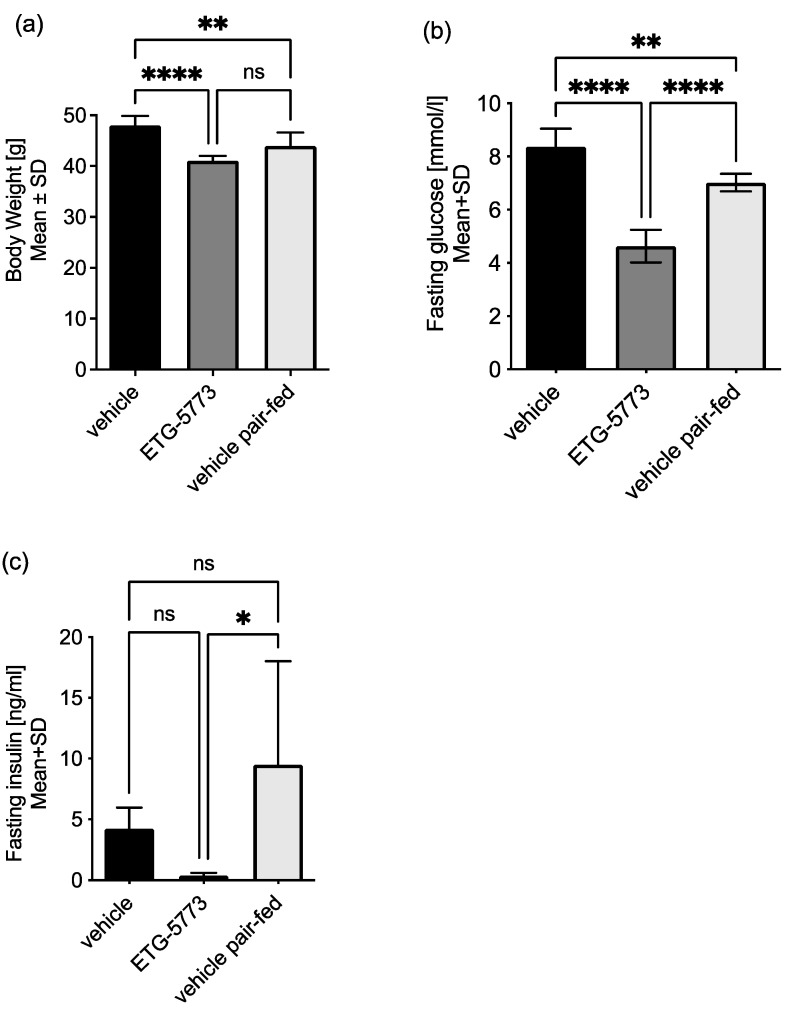
Body weight and glucose regulation in DIO mice treated with ETG-5773 for 7 days. (**a**) Body weight; (**b**) fasting blood glucose concentrations; (**c**) fasting serum insulin concentrations. Animals were treated orally twice a day with 15 mg/kg ETG-5773 or with the corresponding vehicle or vehicle in the pair-fed group. * *p* < 0.05, ** *p* < 0.01, **** *p* < 0.0001 as indicated, ns: non-significant. ANOVA mean SD, *n* = 6.

***Metabolic effects of seven-day ETG-5773 treatment in diet-induced obesity.*** We conducted an additional study in DIO mice for further mechanistic analysis of hepatic energy metabolism. Over a seven-day period, we treated mice twice daily with 15 mg/kg ETG-5773. We also studied vehicle-treated mice and a pair-fed vehicle control group. Overall, seven days of treatment recapitulated most of the metabolic actions observed with 28 days of treatment as shown in Figure 6. Body weight was significantly lower in the treatment group compared to the vehicle control group (−15%, *p* < 0.0001) and was numerically reduced in the pair-fed vehicle group (−7%, *p* = 0.052). There was also a 36% reduction in the fasting blood glucose level (*p* < 0.0001), a >95% reduction in the fasting serum insulin (*p* < 0.05), and a >95% reduction in the homeostatic model assessment for insulin resistance (HOMA-IR, *p* < 0.05) compared to the pair-fed vehicle control. A comparison of the ETG-5773-treated animals with the vehicle group showed reduced fasting blood glucose (−45 %, *p* < 0.0001) and numerically reduced levels of fasting serum insulin (−91 %, *p* = 0.4) and HOMA-IR (−95%, *p* = 0.24). However, the seven-days treatment period was not sufficient to significantly reduce liver fat.

**Figure 7:**
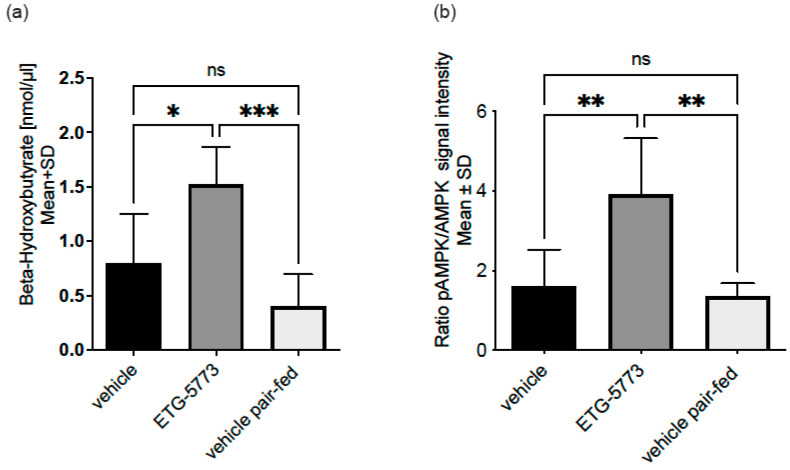
Energy metabolism biomarkers in the DIO mouse model treated with ETG-5773 for 7 days. (**a**) β-hydroxybutyrate (β-HBA) levels measured in an ELISA; (**b**) adenosine monophosphate-activated protein kinase (AMPK) activation levels measured in a Western blot and normalised for actin. Animals were treated orally twice a day with 15 mg/kg ETG-5773 or with the corresponding vehicle or vehicle in the pair-fed group. * *p* < 0.05, ** *p* < 0.01, *** *p* < 0.001 as indicated ns: non-significant. ANOVA mean SD, *n* = 6. Corresponding western blot data are shown in Figure S5.

To understand whether treatment with ETG-5773 also increased hepatic lipid oxidation, we investigated plasma β-hydroxybutyrate (β-HBA), a marker of hepatic lipid oxidation. As shown in Figure 7a, the plasma β-HBA concentrations were significantly increased in treated animals compared to the pair-fed control group (*p* < 0.001) as well as the vehicle control group (*p* < 0.05), suggesting an increase in hepatic lipid oxidation with ETG-5773. Hepatic *de novo* lipogenesis and hepatic lipid oxidation are coordinated by adenosine monophosphate-activated protein kinase (AMPK). Figure 7b indicates the difference in the ratio of phosphorylated AMPK to total AMPK in the livers of the ETG-5773-treated animals compared to the vehicle and pair-fed vehicle rerated animals measured in a Western blot assay. The results are consistent with AMPK activation in treated animals (*p* < 0.01) compared to both control groups.

## 3. Discussion

The aim of our study was to profile the biological activity of the novel selective NaCT (INDY, SLC13A5) inhibitor ETG-5773. In DIO mice, treatment with a moderate ETG-5773 dose twice daily over 28 days decreased body weight, and improved glucose tolerance and insulin sensitivity, as indicated by a reduction in the glucose excursion during the IPGTT and a decrease in the fasting insulin levels by 70%. These metabolic improvements were associated with decreased hepatic steatosis, as liver triglycerides were reduced by 25%. Moreover, key lipogenic genes were downregulated and hepatic lipid oxidation was increased. Plasma cholesterol was not affected and plasma triglycerides slightly increased. Possibly, the capacity of the liver for lipid clearance may be limited such that longer treatment durations are required to improve the circulating lipoprotein profiles. Our data suggest that the beneficial effects are mediated, at least in part, by the activation of hepatic AMPK phosphorylation. This nicely reflects the metabolic changes observed in whole-body *Indy* (*Nact)* (−/−) knockout mice [13,18], as well as mice and rats with selective inducible knock down of Nact (Indy) in the liver [15,16]. We tested ETG-5773’s actions in vitro for its suitability as a selective, cross-species active, small molecule NaCT transport inhibitor. The compound showed a nanomolar inhibitory activity, high selectivity for NaCT against NaDC3 (more than 100-fold) and was greater than 100-fold more active than the structurally different substrate analogue PF-06649298, the only selective small molecule inhibitor so far tested in vivo. Furthermore, the high in vitro activity of ETG-5773 on transport function (IC_50_ 160 nM) translated to downstream physiological changes in fatty acid synthesis with an IC_50_ of 1 µM. ETG-5773 is slightly less active in vitro than the human specific NaCT inhibitor BI01383298. However, its equipotent activity on mouse Nact makes it a valuable tool in studying Nact pharmacology in animal models, which is an important prerequisite for drug development. For example, further studies in rodent models could be conducted to assess the influences of ETG-5773 on liver fibrosis or other metabolic readouts [25].

In a pilot in vivo seven-day tolerability study, we tested a broad dosing range of ETG-5773 between 25 and 125 mg/kg twice daily to select the best dose for long-term study. The study showed an effect on food intake which may be non-target related because such a response was not observed in *Indy* (−/−) knockout mice [13]. Indeed, a separate seven-day tolerability study in *Indy* (−/−) knockout mice showed similar changes in feeding behaviour. The potential for off-target effects on feeding behaviour provided the rational for including pair-fed control groups. Based on these studies, we selected a moderate ETG-5773 dose of 15 mg/kg twice daily for follow up studies, which was well tolerated and pharmacologically active. In fact, pharmacokinetic studies suggested that the dosing regimen provided effective coverage in vivo.

Our results show that, in DIO mice, four weeks of the 15 mg/kg ETG-5773 inhibitor twice-daily treatment improved major components of diet-induced metabolic syndrome including obesity, hepatic steatosis, glucose intolerance, and insulin resistance. Body composition analysis showed relative reductions in fat mass and increased skeletal muscle mass. Except for liver triglycerides, these improvements were evident within seven days of treatment.

ETG-5773 treatment in DIO mice increased hepatic lipid oxidation and reduced *de novo* lipogenesis. β-HBA is an intermediate produced in liver mitochondria mainly from the oxidation of long chain fatty acids. AMPK, a master regulator of energy metabolism, is activated when cellular biochemical energy is reduced, and thus is activated by AMP and inhibited by ATP [26,27]. In the liver, AMPK promotes mitochondrial biogenesis and insulin sensitivity, thereby inhibiting fatty acid synthesis and stimulating fatty acid oxidation.

An analysis of lipogenesis genes revealed *Srebf1* and *Scd1* mRNA downregulation on ETG-5773. SREBF1, also known as sterol regulatory element-binding protein 1 (SREBP-1), the master regulator for most lipogenesis genes, is typically upregulated with high-fat feeding [28]. SCD1, the rate-limiting enzyme in monounsaturated fatty acid synthesis, is also upregulated with high-fat feeding and promotes liver fat deposition [29]. Other lipogenesis genes were also downregulated, such as *Acc1*, *Acly*, and *Fasn*. Taken together, these results indicate that reduced lipogenesis gene expression together with increased lipid oxidation, both controlled by AMPK, attenuated liver fat deposition, at least in part.

These findings resemble observations in *Indy* (−/−) knockout mice on a high-fat diet, suggesting that small molecule inhibitor treatment with ETG-5773 recapitulates the metabolic phenotype associated with genetic *Nact* deletion [13] as well as RNA interfering approaches in mice and rats [15,16].

For the transition from animal studies to clinical testing, NaCT species differences need to be considered. For example, besides different tissue expression patterns, the mouse transporter exhibits a high affinity but low capacity, whereas the human transporter has a low affinity but high capacity for citrate [19]. The differences in substrate affinity may be less important for a non-competitive inhibitor such as ETG-5773 than for substrate inhibitors. ETG-5773’s non-competitive mode of action also means that NaCT inhibition will be less affected by the plasma citrate concentrations. This property differentiates ETG-5773 from PF-06649298 and the related compound PF-06761281, which feature an increased inhibitory potency with increasing citrate concentrations [30]. Additionally, the human selective inhibitor BI01383298 binds irreversibly to NaCT [22]. This competitive action and irreversible binding may complicate dose-finding in human beings. Therefore, ETG-5773 may have advantages, both for preclinical testing and for clinical applications, compared with previous compounds. ETG-5773’s superior specificity for NaCT is another advantage. In contrast, PF-06761281 showed significant activity on the closely related transporters NaDC1 (gene symbol *SLC13A2*) and NaDC3 (gene symbol *SLC13A3*), leading to increased urinary calcium excretion [24], which may have unfavourable effects on bone health and kidney stone formation.

PF-06649298 and PF-06761281, more active but less selective analogues, have been tested in vivo in a DIO model, revealing significant beneficial effects on glucose handling, but only a weak effect on hepatic lipids [23,31]. Other compounds, such as Metformin or Curcumin, have shown beneficial effects in the DIO model and were proposed to act on the NaCT transport function and expression alongside many other pathways. However, high doses of these treatments are typically needed for a pharmacological effect and the primary target may be different from NaCT [32,33].

Another important consideration when transitioning across preclinical and clinical development is that NaCT is predominately but not exclusively expressed in the liver. NaCT is also expressed in neurons affecting neurotransmitter synthesis [34]. Loss of function mutations in NaCT have been implicated in rare autosomal recessive epilepsies known as early infantile epileptic encephalopathy-25 [35]. There have been concerns that NaCT inhibitors crossing the blood–brain barrier might have detrimental effects on neurotransmitter synthesis [34]. ETG-5773 shows some blood–brain barrier penetration with detectable but 30-fold lower concentrations in the brain than in liver. Moreover, *Indy* (−/−) knockout mice only showed a very mild neuronal phenotype [13]. However, others even showed beneficial effects on neuronal *Indy* (−/−) knockout in mice [36].

This study has some limitations. The action of ETG-5773 was only tested in a widely used DIO model and was not compared head-to-head to other inhibitors with different modes of action. The DIO mouse model displays hepatic steatosis without inflammation, fibrosis, or cirrhosis. Further studies are needed to determine whether ETG-5773 is beneficial once hepatic disease has progressed to NASH. Moreover, additional studies are required to determine whether NaCT inhibition acts synergistically with other drugs that are currently in clinical development for NASH, such as GLP1, thyroid hormone receptor (THR) β, farnesoid X receptor (FXR), or PPAR agonists. Moreover, we used a small molecule compound to prove our hypothesis that a therapeutic approach is efficient and safe in diet-induced metabolic disease. Later strategies may also encompass gene therapy, including viral vector delivery and Cripsr-Cas9 guided strategies. This approach will guarantee a more liver-specific effect. Additionally, expression analyses using single cell RNA sequencing as well as metabolic profiling of liver and plasma samples may advance our understanding regarding the molecular mode of action in liver diseases.

In summary, our study proves the concept that inhibition of NaCT-mediated transport is a viable strategy to treat obesity, insulin resistance, and hepatic steatosis. We showed the in vivo efficacy of ETG-5773, the first selective, cross-species active, non-competitive, non-substrate-like NaCT inhibitor, and further validated this transporter as an attractive therapeutic target for metabolic diseases including MAFLD, NASH, obesity, and type 2 diabetes. In subsequent studies, we will optimise compounds based on ETG-5773 to further improve its pharmacokinetic properties. Further in vivo studies are also needed in MAFLD models with benchmarking to other approaches to identify synergies between targeted pathways and potential combination therapies, which are likely to be required when addressing this complex metabolic disease.

## 4. Materials and Methods

### 4.1. Screening and Selection of the NaCT Inhibitor

We obtained the compound by screening a library containing more than 50,000 highly diverse lead-like compounds from the Evotec Discovery Library (Evotec SE) and progressing initial hit-through-hit expansion and hit-to-lead optimisation phases. Compound library details are described under https://www.evotec.com/en/execute/drug-discovery-services/hit-identification (accessed on 5 August 2022). We used [14C] citrate uptake assays with HEK293 cells overexpressing recombinant human NaCT (HEK-NaCT). For confirmation, we measured citrate uptake using HepG2 cells which endogenously express NaCT. Furthermore, we measured activity on mouse Nact in a similar assay system using HEK293 cells recombinantly expressing murine Nact (HEK-Nact). We then tested the selectivity of selected compounds with another transporter of the same family—NaDC3. Selectivity was determined by a succinate uptake assay for NaDC3 using HepG2 cells or using HEK293 cells overexpressing recombinant human NaDC3. Compounds were considered to be selective when they exhibited an at least five-fold, and preferably 10-fold, higher activity for NaCT than for NaDC3.

### 4.2. Cell Culture

We maintained cells in cell medium using cell culture grade flasks (T175 Greiner). The following media were used: HEK293 cells overexpressing NaCT (HEK-NaCT / HEK-Nact) and the respective control cells: MEM (no glutamine) + 10% FCS, 1 × P/S, 2 mM Glutamax, and cells were cultured in the presence of G418 (800 µg/mL); HepG2: MEM (NEAA, no glutamine) + 10% FCS, 1 × P/S, 2 mM Glutamax, 1 mM sodium pyruvate; HEK293 cells expressing NaDC3 (HEK-NaDC3): DMEM + 10% FBS, 100 U/mL penicillin and 0.1 mg/mL streptomycin and cells were cultured in the presence of 150 µg/µL hygromycin. The selection antibiotic G418 (800 µg/mL) was added during cultivation but not for seeding into assay plates. For splitting, cells were washed with PBS (w/o Ca^2+^, Mg^2+^, phenol red) and detached with trypsin/EDTA. Throughout cultivation, cells were kept sub-confluent.

### 4.3. Citrate and Succinate Uptake Assays

We determined activity on human NaCT by measuring citrate uptake into HepG2 cells, which endogenously expressed NaCT [37] and for mouse Nact, using HEK293 cells recombinantly expressing mouse Nact (HEK-Nact). We cloned the mouse *Slc13a5* cDNA encoding Nact as described previously [13].

For radiometric [14C] citrate uptake assays, we used Cytostar-T plates (Perkin Elmer#RPNQ0166, Waltham, MA, USA). For each assay, we seeded 20,000 HepG2 cells per well on collagen coated 384-well Cytostar-T plates or 5000 HEK293 cells per well on ploy-D-lysine coated 384-well Cytostar-T plates. On the following day, we changed culture medium to assay buffer: 120 mM NaCl, 5.4 mM KCl, 0.8 mM MgSO_4_, 5 mM glucose, 1.8 mM CaCl_2_, 25 mM Hepes, 25 mM MES, pH 6.5 (human) and pH 7.5 (mouse). We preincubated cells with compounds at 37 °C for 20 min, after which we added [14C] citrate (3 µM for HEK cells and 10 µM for HepG2 cells) and incubated cells for 50 min at 37 °C. After cooling down for 15 min at 4 °C, we measured the counts using a Microbeta2 reader system (Perkin Elmer).

For radiometric [14C] succinate uptake assays, we applied HEK cells overexpressing human NaDC3 and measured according to the procedure described previously [38], with a modified incubation buffer of HBSS buffer, supplemented with 20 mM HEPES, pH 7.4 and positive control 2,2-Dimethylsuccinic acid (Sigma-Aldrich, St. Louis, MO, USA).

### 4.4. Fatty Acid Synthesis (FAS) and Viability Assay

We performed the fatty acid synthesis assay in HepG2 cells. We seeded cells into black clear-bottom 96-well plates (Corning 3340 cell plate) 75,000/well and incubated at 37 °C. After 16–24 h, we washed the plates once with 100 µL PBS (+CaCl_2_ +MgCl_2_) and added 50 µL assay medium (RPMI 1860 containing 11 mM glucose, 10 mM HEPES, and 1 nM insulin) per well. Then, we applied test substances in assay medium (10 µL, 5% DMSO) and incubated for 20 min at 37 °C. Thereafter, we added 10 µL assay medium containing [14C] citrate (final concentration 50 µM) and PrestoBlue reagent (1× final concentration; Thermo Fisher Scientific, Waltham, MA, USA) and incubated for 180 min (37 °C, 5% CO_2_). We measured cell viability (ViewLux instrument, Perkin Elmer). We removed medium by vacuum aspiration and washed cells once with 100 µL PBS (+CaCl_2_ +MgCl_2_). We lysed cells with 50 µL lysis buffer (0.1 N NaOH, 0.1 % Triton X-100) and sealed plates with Tape Pads and vortexed. For the saponification reaction, we incubated plates for 16–24 h at 70 °C in a humidified environment. Then, we removed seals and added 200 µL 0.1 N HCl to neutralise the pH. We transferred 150 µL from each into corresponding wells of a 96-well Flash plate (Perkin Elmer) and sealed with TopSeal A (Perkin Elmer). We incubated plates for 4 h at 70 °C and for at least 3 h at room temperature in the dark. We measured [14C] radioactivity (microbeta2, Perkin Elmer). For control samples, we applied 50 mM unlabelled citrate or C75 (Sigma-Aldrich, St. Louis, MO, USA, C5490).

### 4.5. Animals

We performed in vivo testing in DIO mice (for 7- and 28-day treatment), an established model designed to study high-fat-diet-induced hepatic steatosis and associated cardiometabolic traits [39,40]. We established DIO in four-week-old C57BL/6N male mice that were fed a high-fat diet (HFD, 60 kcal% fat, D12492; Research Diets, Inc.; New Brunswick, NJ, USA) until 11 weeks of age.

Mice were housed at a temperature of 22–24 °C, with a day/night cycle of 12/12 h (07:00-19:00–07:00) and had access to the appropriate diet and tap water *ad libitum*. Animals allocated to the pair-feeding group were fed the same amount of food as the corresponding treatment group had consumed the day before. Food was only withdrawn for overnight fasting on the penultimate study day before the section on the final day. We randomised groups of n = 12 for treatment and n = 8 for pair-feeding cage-wise according to random fed blood glucose levels and body weight before the study treatment was started. For all animals, the high-fat diet was started at the same age.

Ethics: The study was conducted according to the Annex III of the Directive 2010/63/EU applying to national specific regulations such as the German law on animal protection and approved by the German authorities (reference number 33.9-42502-04-131167, date of approval: 26.06.2013).

### 4.6. Animal Treatment

We treated DIO mice with the inhibitor or corresponding vehicle (5% DMSO/10% Cremophor^®^ EL/85% water) twice a day at the same time by oral gavage according to their grouping (5, 15, and 50 mg/kg dose groups, and vehicle control groups). During the study, we measured body weight and blood glucose. We measured glucose concentrations in tail tip blood samples with the “Accu-Chek^®^ Performa” glucometer (Roche, Basel, Switzerland, range <33.3 mmol/L). We determined random fed blood glucose and fasting blood glucose after overnight fasting.

An intraperitoneal glucose tolerance test was performed after an overnight fasting period. Mice were injected with 1g/kg glucose and blood glucose was measured over a period of 180 min.

At the end of study day 28, we obtained blood and liver samples. In anesthetised animals, we collected retro-orbital blood samples in non-coated tubes (Sarstedt, Inc., Nümbrecht, Germany). Then, we sacrificed animals by cervical dislocation. We dissected and weighed the livers and immediately froze liver samples in liquid nitrogen for further analysis.

For serum sample generation, we centrifuged blood at 4000× *g* for 10 min and dispensed serum samples into a 110 µL plus an additional aliquot. Samples were stored at −80 °C. For Piccolo^®^ system (Abaxis, Inc., Union City, CA, USA) analysis (lipid panel including triglycerides and cholesterol), we used a 110 µL serum aliquot.

The frozen tissue from liver samples were used to determine the triglyceride content via triglyceride assay according to the manufacturer’s protocol (Sigma-Aldrich, Cat.No.TR0100) using glycerol as a standard (Sigma-Aldrich, Glycerol G7793, St. Louis, MO, USA).

### 4.7. Body Composition Analysis

We determined body composition in mice using the non-invasive Bruker Minispec Instrument MQ 10 NMR Analyser (Bruker, Billerica, MA, USA). We placed mice in a clear, plastic cylinder and kept them immobile by inserting a tight-fitting plunger into the cylinder. For scanning, the tube was placed in the sample chamber for approximately one minute. Body composition was calculated and related to whole body weight.

### 4.8. Lipogenesis Gene Expression Analysis

We isolated total RNA from individual liver samples from all animals in 1.5 mL Trizol reagent (Life Technologies, Carlsbad, CA, USA) at 4 °C according to the manufacturer’s instructions. Then, we purified total RNA utilising the RNeasy Mini Kit (Qiagen, Hilden, Germany) with DNase-treatment to completely remove genomic DNA according to the instructions of the manufacturer. Subsequently, RNA was reverse transcribed using Superscript II RnaseH Reverse Transcriptase (Life Technologies) and subjected to Taqman analysis using the Taqman Fast Advanced Master Mix (Applied Biosystems, Weiterstadt, Germany). The Mix contains AmpliTaq^®^ Fast DNA Polymerase, AmpErase UNG, dNTPs with dUTP, passive reference Rox and optimised buffer components. Quantitative PCR was performed in the Quantstudio 7 Flex System (Applied Biosystems, Weiterstadt, Germany). The relative expression of genes was analysed by the ΔΔCt value method using 18s ribosomal RNA as endogenous control.

We used the following primer probe pairs to amplify the *SREBF1* gene: Forward primer 5′-GGCACTAAGTGCCCTCAACCT-3′; Reverse Primer 5′-GCCACATAGATCTCTGCCAGTGT 3′; Probe 5′-TGCGCAGGAGATGCTATCTCCA 3′. For the *SCD1* gene the Mm00772290_m1 Taqman Assay kit was used (Thermo Fisher Scientific, Waltham, MA, USA).

### 4.9. Beta-Hydroxybutyrate ELISA

We determined serum beta-hydroxybutyrate with a Beta-Hydroxybutyrate Assay kit, (Sigma-Aldrich, Cat.No. MAK041, St. Louis, MO, USA) following the manufacturer’s instructions. The sample was undiluted, and the volume was 5 µL.

### 4.10. AMPK Analysis

We performed Western blots as described previously [13]. Deviating from that protocol samples were heated for 5 min at 95 °C and blotting time was 1 h at 20 volts. For target protein detection, AMPK alpha (D63G4) Rabbit mAb (Cell Signaling Technology, Inc., Danvers, MA, USA; Cat. No. #5832/3; working dilution 1:1000) and Phospho-AMPK alpha (Thr172) (40H9) Rabbit mAb (Cell Signaling Technology, Inc.; Cat. No. #2535/16; working dilution 1:1000) were used; gel loading was controlled by anti-beta-actin (Sigma-Aldrich, Cat. No. A2228). We detected signals with the SuperSignal West Dura Extended Duration Substrate (Thermo Fisher Scientific; Cat. No. 34076, Waltham, MA, USA).

### 4.11. Plasma Lipid Analysis

VetScan^®^ system was used for plasma lipid analysis and 110 µL-aliquot of lithium-heparin blood plasma was collected. For this purpose, the blood samples were taken by retro-orbital bleeding and collected in tubes coated with lithium-heparin (Sarstedt, Inc.). After incubation at RT for at least 10 min, samples were centrifuged at 4000× *g* for 10 min, and the supernatant was then transferred to a new tube and stored at −80 °C.

### 4.12. Compounds and Cell Lines

PF-06649298, BI01383298 and ETG-5773 were synthesised by Evotec. For ETG-5773, different batches were used for our studies. All compound batches were tested for activity in citrate uptake assay in HepG2 and mNaCT HEK293 and showed comparable activity. The following were used for the 28-day treatment: EV-AO6978-002; 7-day treatment: EV-AO6975-002; and in vitro studies: EV-AO6180-001.

The HEK293 cell line was obtained from DSMZ and transfected with vectors for human and mouse NaCT/SLC13A5 as described in Birkenfeld et al. 2011 [13]). HepG2 cells were obtained from ATCC.

### 4.13. Statistical Analysis

Data are presented as mean ± standard deviation (SD). Multiple comparisons were performed by one- or two-way analysis of variance (ANOVA) and Tukey’s post hoc analysis. Prism 9 (GraphPad Software Inc., San Diego, CA, USA) was used for statistical analysis and *p* < 0.05 was considered significant.

## 5. Conclusions

Our study provides the first report of a cross species-active, non-competitive, small molecule inhibitor for NaCT. Treatment of DIO mice with the ETG-5773 inhibitor over four weeks improved body weight, liver fat, body composition, and glucose metabolism, indicating improved insulin sensitivity. These findings pave the way for a small-molecule NaCT-mediated transport inhibitor as a potential treatment for patients with MAFLD and NASH. In light of our findings and previous data [13,14,15,16,18], the inhibition of NaCT seems to be an attractive target as a new approach to address the complexity of this highly prevalent disease.

## Figures and Tables

**Table 1:** In vitro data for the effect of the inhibitor ETG-5773 on cell lines expressing human and mouse transporters.

	[14C] Substrate Uptake			Fatty Acid Synthesis	Cytotoxicity
Cell line	HepG2	HEK293	HEK293	HepG2	HepG2
Species	Human	Mouse	Human	Human	Human
Substrate	Citrate	Citrate	Succinate	Citrate	-
Transporter	NaCT	Nact	NaDC3	NaCT	-
IC_50_	160 nM	180 nM	>20 µM	1 µM	>50 µM

**Table 2:** Comparison of ETG-5773 with other known inhibitors. Compound structures are shown in Figure S1.

IC_50_ Measurements	ETG-5773	PF-06649298	BI01383298
HepG2 citrate uptake (human)	160 nM	50 µM	78 nM
HepG2 fatty acid synthesis	1 µM	39 µM	30 nM
HEK293 citrate uptake (mouse)	180 nM	6.6 µM	>50 µM

## Data Availability

All data for this study are provided in the manuscript and in supplemental data.

## Supplementary Materials

The following supporting information can be downloaded at: https://www.mdpi.com/article/10.3390/metabo12080732/s1, Figure S1: Molecular structures of tested compounds (a) ETG-5773 (b) PF-06649298 (c) BI01383298; Figure S2: Example data for citrate uptake assay of ETG-5773 (filled circles) with a significant less active analogue ETG-5436 (open circles) considered as a negative control; Figure S3: Plasma lipid analysis of DIO mice after the 28-day treatment period. (a) plasma cholesterol, (b) plasma triglycerides. Animals were treated orally twice a day with ETG-5773 at 15 mg/kg or with the corresponding vehicle or vehicle in the pair-fed group. ** *p* < 0.01 as indicated, ns: non-significant. ANOVA mean SD, *n* = 12 vehicle and ETG-5773, *n* = 8 pair-fed; Figure S4: mRNA expression levels from additional genes involved in hepatic lipogenesis in liver tissue of DIO mice after the 28-day treatment period. Animals were treated orally twice a day with ETG-5773 at 15 mg/kg or with the corresponding vehicle or vehicle in the pair-fed group: (a) mRNA expression of ATP citrate lyase (*ACLY*) gene; (b) mRNA expression of Acetyl CoA carboxylase 1 (*ACC1) gene*; (c) mRNA expression of fatty acid synthetase (FASN) * *p* < 0.05, ** *p* < 0.01 as indicated, ns: non-significant. ANOVA mean SD, *n* = 12 vehicle and ETG-5773, *n* = 8 pair-fed; Figure S5: Western blot for quantifying adenosine monophosphate-activated protein kinase (AMPK), Thr172 phosphorylated AMPK, and ß-actin. Animals were treated orally twice a day with ETG-5773 at 15 mg/kg or with the corresponding vehicle or vehicle in the pair-fed group, *n* = 6 for all groups.
