# Supplementary material for: A Novel and Cross-Species Active Mammalian INDY (NaCT) Inhibitor Ameliorates Hepatic Steatosis in Mice with Diet-Induced Obesity
Source: Metabolites. 2022 Aug 8;12(8):732. doi: 10.3390/metabo12080732 (PMC9413491; doi:10.3390/metabo12080732)
Supplement: Supplementary file 1 [file metabolites-12-00732-s001.zip › metabolites-1792315 SM.pdf]

(a) (b) (c)

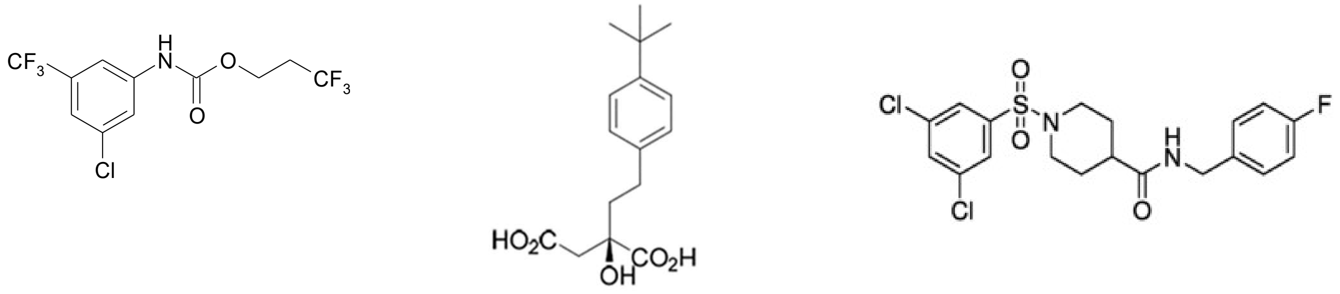

Figure S1. Molecular structures of tested compounds (a) ETG-5773, (b) PF-06649298 and (c) BI01383298.

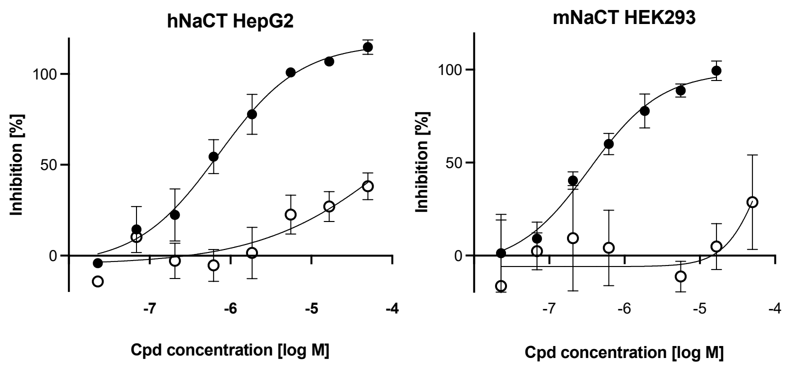

Figure S2. Example data for citrate uptake assay of ETG-5773 (filled circles) with a significant less active analogue ETG-5436 (open circles) considered as a negative control.

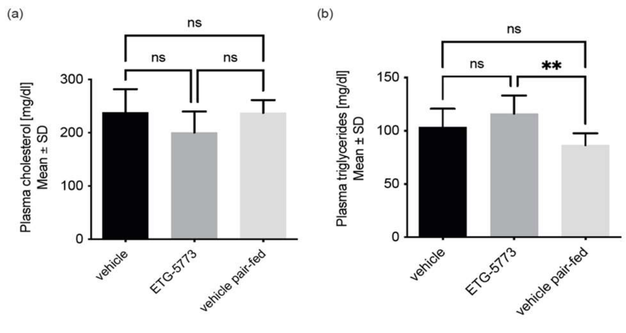

Figure S3. Plasma lipid analysis of DIO mice after the 28-day treatment period. (a) plasma cholesterol, (b) plasma triglycerides. Animals were treated orally twice a day with ETG-5773 at 15 mg/kg or with the corresponding vehicle or vehicle in the pair-fed group. ** p<0.01 as indicated, ns: non-significant. ANOVA mean SD, n=12 vehicle and ETG-5773, n=8 pair-fed

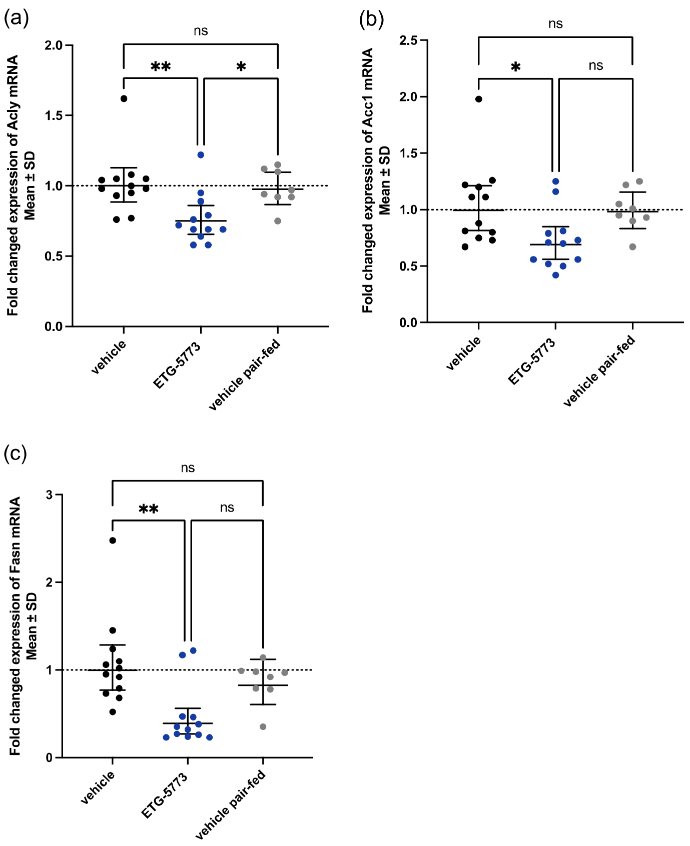

Figure S4. mRNA expression levels from additional genes involved in hepatic lipogenesis in liver tissue of DIO mice after the 28-day treatment period. Animals were treated orally twice a day with ETG-5773 at 15 mg/kg or with the corresponding vehicle or vehicle in the pair-fed group: (a) mRNA expression of ATP citrate lyase (*ACLY*) gene; (b) mRNA expression of Acetyl CoA carboxylase 1 (*ACC1) gene*; c) mRNA expression of fatty acid synthetase (FASN) * p<0.05, ** p<0.01 as indicated, ns: non-significant. ANOVA mean SD, *n* = 12 vehicle and ETG-5773, n=8 pair-fed

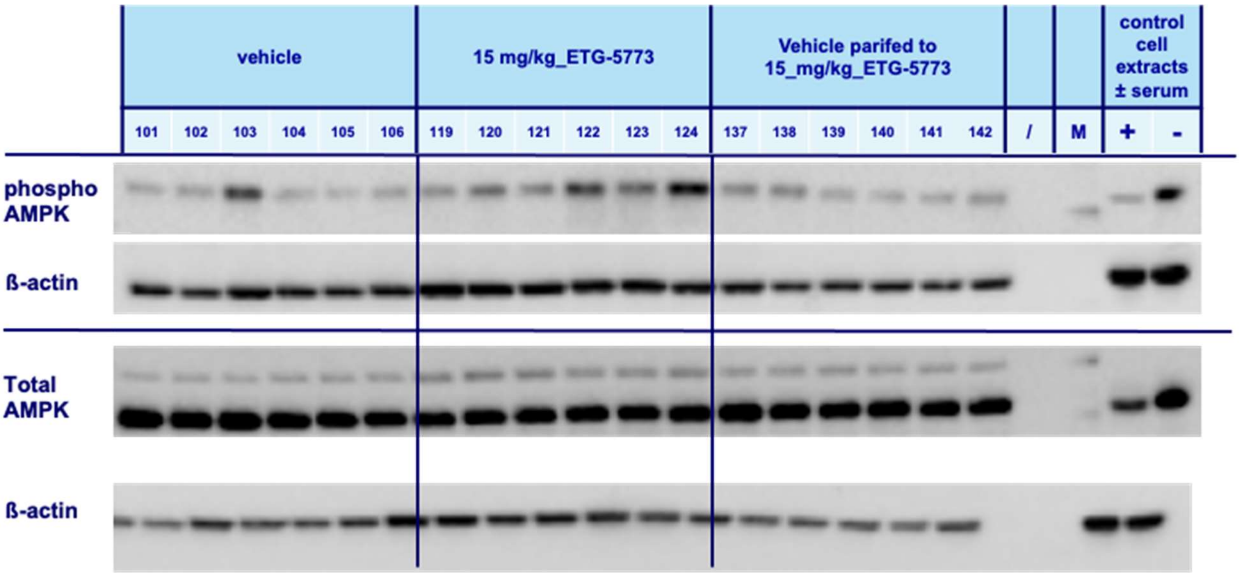

Figure S5. Western blot for quantifying adenosine monophosphate-activated protein kinase (AMPK), Thr172 phosphorylated AMPK and ß-actin. Animals were treated orally twice a day with ETG-5773 at 15 mg/kg or with the corresponding vehicle or vehicle in the pair-fed group, n=6 for all groups.
